# Circadian Rhythm of Body Color Change in the Juvenile Chinese Giant Salamander (*Andrias davidianus*) Under Different Photoperiods

**DOI:** 10.3390/ani15111526

**Published:** 2025-05-23

**Authors:** Yifang Zhang, Ziteng Wang, Qinghua Luo, Honghui Li, Pei Wang, Jiuxiang Wang, Dafeng Li, Wentao Wang, Kangle Yuan, Yan Zhou, Shouliang Luo, He Tian

**Affiliations:** 1 Hunan Engineering Laboratory for Chinese Giant Salamander’s Resource Protection and Comprehensive Utilization, School of Biological Resources and Environmental Sciences, Jishou University, Zhangjiajie 427000, China; zyf33629@163.com (Y.Z.); 16681982610@163.com (Z.W.); wangpei0229@126.com (P.W.); ldf203540@163.com (D.L.); 13610513561@163.com (Y.Z.); luosl2021@163.com (S.L.); tianhe970808@126.com (H.T.); 2 Hunan Engineering Technology Research Center for Amphibian and Reptile Resource Protection and Product Processing, College of Biological and Chemical Engineering, Changsha University, Changsha 410022, China; lee19890925@163.com (H.L.); wangjiuxiang@outlook.com (J.W.); 13451324170@163.com (W.W.); 13028648168@163.com (K.Y.)

**Keywords:** Amphibian, reflectance spectra, cosinor rhythm modeling, UV photoprotection, physiological color change

## Abstract

The circadian rhythm of an animal’s body color change is crucial for its survival and adaptation to the environment, with photoperiod serving as a primary “zeitgeber” that significantly affects the circadian rhythm of color change. To explore the circadian body color change in juvenile Chinese giant salamanders (*Andrias davidianus*), four distinct photoperiod regimens were established, including the DD group (constant darkness), the LD12:12 group (12 h photophase, 12 h scotophase), the LD16:8 group (16 h photophase, 8 h scotophase), and the LD8:16 group (8 h photophase, 16 h scotophase). Reflectance spectra of dorsal skin were measured every 4 h over a 24 h cycle, with data collected every other day for three consecutive days, totaling 72 h. Reflectance spectra were converted into colorimetric variables to analyze circadian patterns. The results demonstrated that juvenile *A. davidianus* exhibited highly significant circadian rhythmicity under different photoperiods, with their body color becoming darker during the photophase (mean brightness: 14.650 ± 0.629 to 16.385 ± 1.301) and lighter during the scotophase (mean brightness: 16.473 ± 0.875 to 34.422 ± 2.692). Photoperiod alterations significantly affected the rhythm parameters (mesor, amplitude, and acrophase) of mean brightness variation. Compared with the LD12:12 group, the LD16:8 group (extended photophase) reduced rhythm mesor by 1.415, decreased amplitude by 0.988, and induced a 1.510 h acrophase delay. Conversely, the LD8:16 group (extended scotophase) increased rhythm mesor by 2.141 and amplitude by 2.919. These findings indicate that the circadian rhythm of body color change in juvenile *A. davidianus* is coordinately regulated by endogenous circadian rhythms and exogenous photoperiodic signals. The findings of this study provide a theoretical foundation for the conservation and management of *A. davidianus*.

## Simple Summary

Certain animals, including crabs, frogs, and lizards, change their body color to match daily light cycles, which is crucial for their survival. This study investigated how juvenile Chinese giant salamanders (*Andrias davidianus*), an endangered species, adjust their body color between day and night under different artificial light–dark cycles. We exposed the juveniles to four different light conditions: constant darkness, equal light–dark cycles (12 h light and 12 h dark), longer days (16 h light and 8 h dark), and longer nights (8 h light and 16 h dark). By measuring their body color every four hours for three days, we found that these juveniles naturally darken during light periods and lighten in the dark. Their body color changes follow a daily rhythm, which adjusts when days or nights are made longer. For example, longer days caused weaker color changes, while longer nights led to stronger changes. These results show that the juveniles’ color patterns rely on both their internal body clock and external light cues. This research helps us protect this rare species by revealing how they adapt to environmental change. By studying these rhythms, we can better support wildlife conservation and understand how animals maintain their survival strategies in a changing environment.

## 1. Introduction

The ability of animals to modulate color through brightness or hue adjustments in response to specific stimuli holds significant biological implications for environmental adaptation and evolution. This phenomenon is particularly prominent in ectothermic animals, including crustaceans [1], cephalopods [2], fishes [3,4], amphibians [5], and reptiles [6]. Amphibian body color changes exhibit both aperiodic and periodic characteristics [7]. Aperiodic body color changes are triggered by factors such as ontogenetic development [8,9], predation risk [10], reproductive requirements [11,12], and sudden changes in environmental factors (e.g., temperature, light, and substrate color) [13,14]. Periodic body color changes arise from exogenous adaptation to cyclic environmental factors, including diurnal/seasonal light and temperature cycles [15,16] and seasonal substrate color changes [17] among others, or are simultaneously regulated by an internal biological clock that remains self-sustaining in the absence of environmental cues [18]. As a principal zeitgeber, light–dark cycles entrain multiple circadian behaviors in amphibians, such as locomotor activity, respiration, mating, and feeding [19,20,21,22].

Research on circadian body color changes has primarily focused on crustaceans, whose circadian rhythm of body color changes is regulated by neurosecretory hormones to provide UV protection and camouflage benefits [7,23,24]. While circadian behaviors [25] and body color changes [5] have been reported in amphibians, the circadian rhythm of body color changes and their molecular mechanisms have not been elucidated. To date, only the yellow cururu toad (*Rhinella icterica*, formerly *Bufo ictericus*) has been reported to exhibit circadian body color changes, darkening during daytime and lightening at night [26]. This rhythmic pattern may function to protect them from UV radiation during daytime and conserve energy at night [27].

The Chinese giant salamander (*Andrias davidianus*) belongs to the class Amphibia, order Caudata, family Cryptobranchidae, and genus *Andrias*. Cryptobranchidae is one of the most ancient extant amphibian lineages, with fossil records dating back to the Middle Jurassic [28]. The genus *Andrias* diverged from its sister genus *Cryptobranchus* during the late Cretaceous, while *A. davidianus* and the Japanese giant salamander (*Andrias japonicus*) diverged in the late Miocene [28]. Traditionally, the Chinese giant salamander was considered a single species (*A. davidianus*). However, recent molecular phylogenetic studies suggest the existence of 5–7 cryptic species within this complex [29,30,31]. To date, four species have been formally recognized: *Andrias davidianus* [32], *Andrias sligoi* [33], *Andrias jiangxiensis* sp. nov. [34], and *Andrias cheni* sp. nov. [35], with *Andrias sligoi* identified as the world’s largest extant amphibian [31]. Since the 1950s, wild populations have experienced drastic declines due to over-acquisition, illegal poaching, and habitat destruction [36]. Consequently, it is classified as a National Grade II Protected Wildlife Species by China’s National Forestry and Grassland Administration (2021) [37] and listed as Critically Endangered by the International Union for Conservation of Nature (IUCN) [38]. Adults *A. davidianus* typically exhibit brown, yellow-brown, or gray-brown color with irregular black/dark brown dorsal–ventral patterns, while juvenile *A. davidianus* display dark brown with black spots [39]. Due to their cryptic lifestyle, systematic studies into their body color change remain absent. Only Liang (2015) described that post-emergence larvae gradually transition from flesh-toned to dark black upon initial light exposure [40]. This study investigates the circadian body color change in juvenile *A. davidianus*, exploring the connection between body color change and biological clock, as well as the adaptation of juveniles to light change, laying the foundation for revealing the physiological and molecular mechanisms underlying the circadian rhythm of body color change in *A. davidianus*. In this study, we aim to (1) demonstrate that the body color change in juvenile *A. davidianus* has a circadian rhythm; (2) confirm the endogenous nature of this rhythmic change; and (3) elucidate how photoperiodic cues entrain body color change rhythm.

## 2. Materials and Methods

### 2.1. Animals

The experimental juvenile giant salamanders were confirmed as *A. davidianus* through consultation with the breeding facility, originating from a single pair of locally sourced parental specimens within a controlled breeding cave, with no history of hybridization with other *Andrias* species. Both the parental giant salamanders and their offspring were sourced from the ecological breeding base of Zhuyuan Giant Salamander Biotechnology Co., Ltd., located in Kongkeshu Township, Sangzhi County, Zhangjiajie City, Hunan Province, China (29°25′56″ N, 110°22′55″ E; altitude: 471 m). Forty 16-month-old juveniles (body weight: 15.89 ± 2.19 g; body length: 14.87 ± 1.03 cm) were acclimated in white polyethylene tanks under laboratory conditions before experimentation. The water temperature was monitored at 12.40 ± 0.86 °C using a temperature data logger (MX2203, HOBO, Onset Computer Corporation, Bourne, MA, USA) with a light–dark cycle of LD12:12 (7:00–19:00 as the photophase and 19:00–7:00 as the scotophase). Full-spectrum illumination approximating solar radiation was provided by a 25W UVB lamp (Exo Terra Reptile UVB 100, Exo Terra, Hagen Inc., Montreal, Canada) suspended 1 m above the tanks, delivering 50~60 lux at the water surface level. Shelters were provided to minimize stress during the photophase. Water was renewed daily, and bloodworms were supplied every other day as dietary provision.

### 2.2. Experiments

From March to April 2024, four photoperiod regimens were implemented: (1) LD12:12 group: simulating natural day–night cycles with a 12 h photophase (07:00–19:00) and 12 h scotophase (19:00–07:00); (2) DD group: 24 h constant darkness; (3) LD16:8 group: 16 h photophase (07:00–23:00) and 8 h scotophase (23:00–07:00); (4) LD8:16 group: 8 h photophase (07:00–15:00) and 16 h scotophase (15:00–07:00). The DD group served to verify the endogenous nature of circadian rhythm of body color change in juvenile *A. davidianus*, while the remaining three groups were designated to examine whether different photoperiods entrain circadian rhythm of body color change in juveniles. The water temperature during the experimental period was monitored at 17.87 ± 2.12 °C using a temperature data logger (MX2203, HOBO, Onset Computer Corporation, Bourne, MA, USA). All photoperiod regimens maintained identical photophase light conditions (intensity and spectrum) to those used during acclimation, with instantaneous transitions between photophase and scotophase. This design standardized light intensity and spectral composition across the groups to isolate the effects of photophase/scotophase duration on circadian body color changes.

Following experimental initiation, juvenile *A. davidianus* were acclimated to their respective photoperiods for 7 days before measurements. Dorsal skin reflectance spectra (200–1100 nm) were acquired using a fiber-optic spectrometer (Avaspec-ULS2048CL-EVO, Avantes, Avantes BV, Apeldoorn, The Netherlands) under controlled conditions. Each 24 h experimental cycle began at 09:00, with measurements taken at 4 h intervals (6 timepoints per cycle). Data acquisition spanned three alternating days, resulting in 18 temporal measurements over 72 h. Reflectance spectra were recorded via AvaSoft software (v8.14, Avantes BV, Apeldoorn, The Netherlands). The light source was warmed up for 20 min before each measurement, and then acquired dark noise and white reference (WS-2, Avantes, Avantes BV, Apeldoorn, The Netherlands) for calibration. During measurements, the reflection probe (FCR-7UVIR400-2-ME, Avantes, Avantes BV, Apeldoorn, The Netherlands) was positioned perpendicular to the dorsal skin surface at a fixed distance of 2 mm, with an integration time of 600 ms. All procedures were conducted in a light-controlled darkroom to eliminate ambient interference.

### 2.3. Statistical Analysis

The spectral data were extracted and exported as TXT files using the accompanying software AvaSoft 8.14. Reflectance spectra (300–700 nm) of the dorsal skin of juvenile *A. davidianus* were imported into the R package pavo (v2.9.0). This wavelength range, standard for animal chromatic analysis, encompasses both human-visible light and near-ultraviolet light perceptible to animals while avoiding noise interference at the front and the end of the spectra, thereby enabling comprehensive and precise chromatic analysis [41]. The spectra were averaged, smoothed, and converted into intuitive colorimetric variables (dimensionless) to measure the body color of juvenile *A. davidianus*. Mean brightness (B2) was defined as the mean relative reflectance over the entire spectral range, while chroma (S1) quantified the relative contribution of a spectral range to the total brightness (B1), subdivided into six parameters related to specific hues: ultraviolet (S1U, 300–400 nm), violet (S1V, 300–415 nm), blue (S1B, 400–510 nm), green (S1G, 510–605 nm), yellow (S1Y, 550–625 nm), and red (S1R, 605–700 nm) [42]. To assess temporal variations in colorimetric variables under different photoperiods, one-way ANOVA was performed using R 4.2.3, identifying statistically significant differences in colorimetric variables across different timepoints.

To detect the circadian rhythm of body color change in juvenile *A. davidianus*, mean brightness was selected as the response variable to characterize overall circadian body color change [15,26,43]. Mean brightness (mean relative reflectance over the entire spectral range) integrates composite reflective properties of all wavelengths, thereby minimizing localized biases or hue-specific responses inherent to singular chromatic parameters (e.g., red, green, blue) and facilitating quantification of holistic circadian patterns. Rhythm analysis was performed on time series of individual mean brightness using the R package circacompare (v0.2.0) to estimate the rhythm significance, mesor (the rhythm-adjusted average of mean brightness), amplitude (half of the difference between the peak and trough), and acrophase (the time at which the rhythm peaks), which are the three core parameters for resolving circadian rhythm [44]. These parameters collectively describe circadian characteristics across three dimensions: base level, fluctuation intensity, and temporal synchronization. For each photoperiod group, rhythmic individuals were analyzed to compare inter-group differences in these circadian parameters.

## 3. Results

### 3.1. Spectral Characteristics of Dorsal Skin of Juvenile A. davidianus Under Different Photoperiods

**Figure 1:**
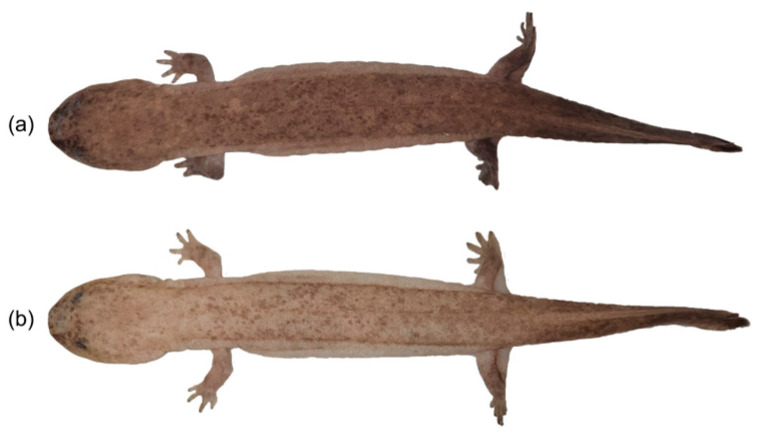
Diurnal–nocturnal comparison of body color in juvenile *A. davidianus*. (**a**) Juvenile *A. davidianus* exhibit dark-brown color during the photophase and (**b**) light-brown color during the scotophase.

**Figure 2:**
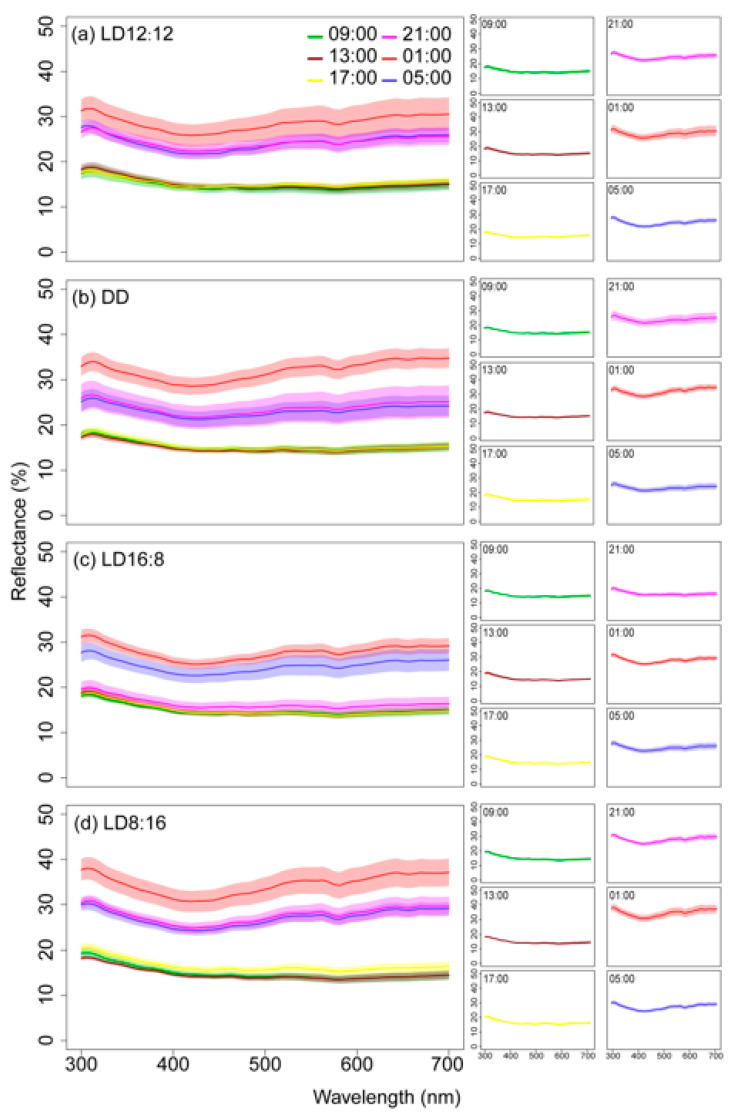
Diurnal–nocturnal comparison of dorsal skin reflectance spectra in juvenile *A. davidianus*. Curves represent average reflectance spectra of dorsal skin in juvenile *A. davidianus* at different timepoints; shadows represent standard deviations.

Juvenile *A. davidianus* appeared dark brown during the photophase and light brown during the scotophase (Figure 1). Across all photoperiod treatments, dorsal skin reflectance spectra during the photophase displayed unimodal curves with a reflection peak at 300–320 nm. Reflectance decreased progressively with increasing wavelength in the short-wave region (300–400 nm) and stabilized beyond 400 nm (400–700 nm). In contrast, scotophase spectra exhibited bimodal curves characterized by dual peaks at 300–320 nm and 650–700 nm. Reflectance declined gradually in the short-wave range (300–400 nm), transitioned to an ascending trend in the mid-wave region (400–650 nm), and plateaued in the long-wave range (650–700 nm) (Figure 2). The results demonstrate that under different photoperiods, the dorsal skin reflectance spectra of juvenile *A. davidianus* differ significantly between photophase and scotophase, exhibiting distinct circadian variations. During the photophase, the spectral profiles were similar across groups, characterized by relatively flat curves with minimal fluctuations. In contrast, scotophase spectra displayed pronounced undulations and greater fluctuations. Among the scotophase of groups, spectral fluctuations were largest in the LD8:16 group, lowest in the LD16:8 group, and intermediate in the LD12:12 and DD groups. Notably, the intensity of spectral undulations was most obvious at 01:00 during the scotophase compared to other timepoints.

### 3.2. Body Color Change in Juvenile A. davidianus Under Different Photoperiods

**Figure 3:**
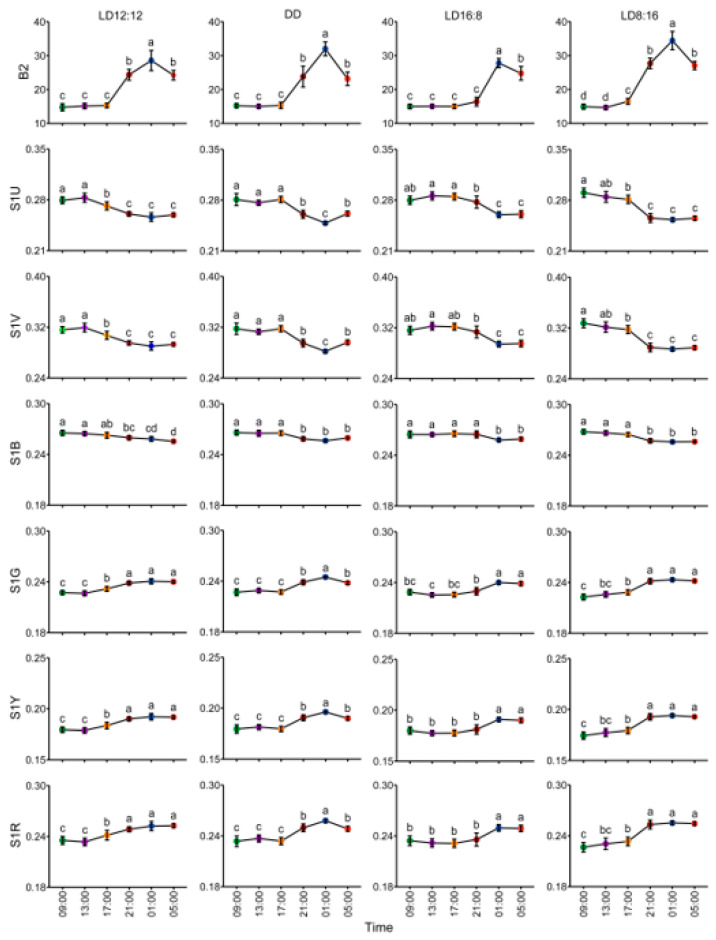
Diurnal–nocturnal differences in body color changes in juvenile *A. davidianus*. Different lowercase letters indicate significant differences (*p* < 0.05).

In the LD12:12 group, one-way ANOVA revealed no significant differences in the colorimetric variable S1B between 17:00 (photophase) and 21:00 (scotophase). However, the S1B values at 09:00 and 13:00 (photophase) were significantly higher than those during scotophase. Conversely, variables B2, S1G, S1Y, and S1R exhibited significantly lower values during photophase compared to scotophase, while S1U and S1V showed the opposite pattern. Within the photophase, B2 and S1B have no significant differences at the different time points. S1U and S1V at 17:00 were significantly reduced compared to 09:00 and 13:00, whereas S1G, S1Y, and S1R increased significantly. No inter-timepoint differences were observed between 09:00 and 13:00 for any variables. Within the scotophase, B2 at 1:00 was significantly higher than at 5:00 and 21:00, and S1B at 5:00 was significantly higher than at 21:00, with no significant differences detected between other timepoints. The remaining colorimetric variables showed no statistically significant temporal variations during the scotophase (Figure 3).

In the DD group, one-way ANOVA revealed significant differences in colorimetric variables between the subjective photophase and scotophase. Specifically, B2, S1G, S1Y, and S1R exhibited significantly lower values during the subjective photophase compared to scotophase, while S1U, S1V, and S1B showed the opposite trend. During the subjective photophase, no temporal variations were observed in any colorimetric variables across measurement timepoints. During the subjective scotophase, S1B demonstrated no significant differences between timepoints. However, B2, S1G, S1Y, and S1R were significantly higher at 01:00 compared to 05:00 and 21:00, whereas S1U and S1V were significantly lower. No inter-timepoint differences were detected between 05:00 and 21:00 for any variables (Figure 3).

In the LD16:8 group, one-way ANOVA revealed significant differences in colorimetric variables between the photophase and scotophase. Specifically, B2, S1G, S1Y, and S1R exhibited significantly lower values during the photophase compared to scotophase, whereas S1U, S1V, and S1B showed the opposite trend. Within the photophase, S1U at 21:00 was significantly lower than at 13:00 and 17:00, while S1V at 13:00 was significantly higher than at 21:00, and S1G at 13:00 was significantly lower than at 21:00. No differences were observed for S1U, S1V, and S1G between other timepoints. The remaining colorimetric variables showed no significant variations across the photophase. During the scotophase, B2 was significantly elevated at 01:00 compared to 05:00, with no differences detected at other timepoints. All other colorimetric variables demonstrated no statistically significant temporal variations in scotophase (Figure 3).

In the LD8:16 group, one-way ANOVA demonstrated significant colorimetric variations between photophase and scotophase. B2 was significantly lower during the photophase compared to scotophase. S1U and S1V showed significantly higher values at 09:00 (photophase) than during the scotophase, and at 13:00 (photophase) compared to 21:00, 01:00, and 05:00 (scotophase), but showed no difference relative to 17:00 (scotophase). Similarly, S1B was elevated during the photophase compared to 21:00, 01:00, and 05:00 (scotophase), with no significant difference at 17:00 (scotophase). Conversely, S1G, S1Y, and S1R were decreased significantly at 09:00 (photophase) versus at the scotophase and at 13:00 (photophase) compared to 21:00, 01:00, and 05:00 (scotophase), but did not differ from 17:00 (scotophase). Within the photophase, no temporal variations were observed in any colorimetric variables. During the scotophase, S1U, S1V, and S1B were significantly higher at 17:00 compared to 21:00, 01:00, and 05:00, while B2, S1G, S1Y, and S1R were significantly lower at 17:00 than at other scotophase timepoints. Notably, B2 was significantly elevated at 01:00 relative to 17:00, 21:00, and 05:00. No significant differences were observed in colorimetric variables between the remaining timepoints (Figure 3).

The observed differences in diurnal–nocturnal chromatic variations in juvenile *A. davidianus* revealed different body colors between the photophase and scotophase across the photoperiod treatments. The colorimetric variables B2, S1G, S1Y, and S1R were predominantly lower during the photophase compared to scotophase, while S1U, S1V, and S1B exhibited the inverse pattern. During the photophase, body color remained stable with no significant differences among variables. In contrast, the scotophase displayed pronounced body color variations, particularly at specific timepoints (e.g., 01:00). These nocturnal dynamics followed a biphasic trend: gradual lightening during the early scotophase, reaching lightest at the mid-dark phase, followed by progressive darkening toward dawn.

### 3.3. Circadian Rhythm and Differences in Body Color Change in Juvenile A. davidianus Under Different Photoperiods

All 40 juvenile *A. davidianus* in the LD12:12, DD, LD16:8, and LD8:16 groups showed highly significant circadian rhythms in mean brightness, with the rhythm period defaulting to 24 h. The mesor of these rhythms ranged from 18.980 to 22.536, with the LD16:8 group showing the lowest value and the LD8:16 group the highest. The amplitude of these rhythms ranged from 6.612 to 10.520, with the LD16:8 group being the smallest and the LD8:16 group being the largest. The acrophase was significantly delayed in the LD8:16 group (17.416 h, approximating 02:25), whereas other groups exhibited similar peak times (15.778~15.908 h, approximating 00:47~00:54) (Table 1). These results confirm that body color changes in juvenile *A. davidianus* maintain highly significant circadian rhythms under all tested photoperiods, with the LD8:16 group displaying the strongest rhythmic intensity and longest delay in the peak time, the LD16:8 group the weakest, and the LD12:12 and DD groups intermediate values between them.

**Figure 4:**
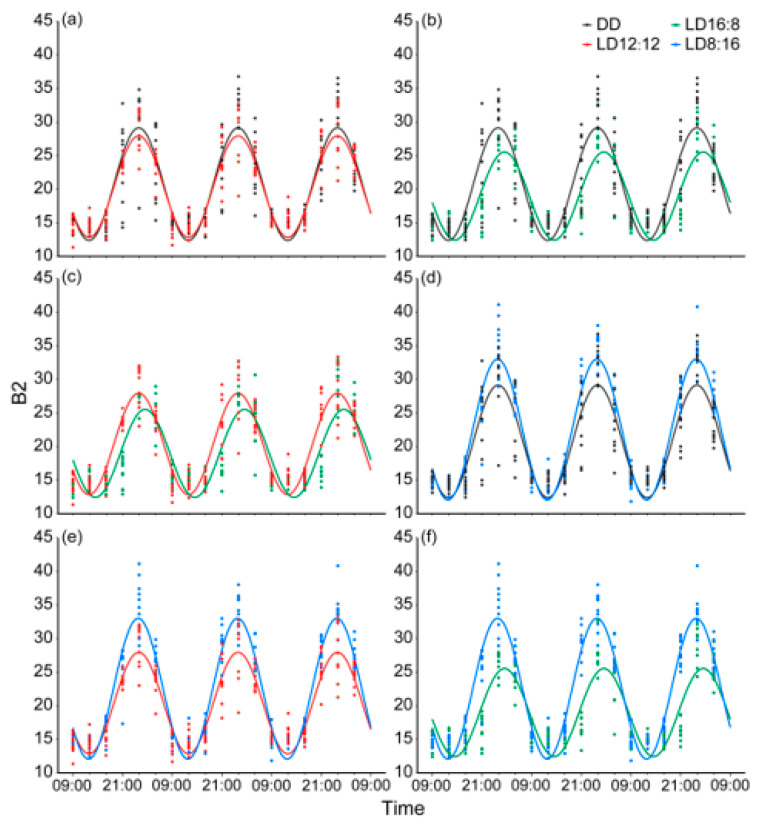
Differences in circadian rhythms of mean brightness variation between photoperiod groups. (**a**) No significant differences in mesor, amplitude, or acrophase between the DD group and LD12:12 group (*p* > 0.05); (**b**) Highly significant differences in mesor, amplitude and acrophase between the DD group and LD16:8 group (*p* < 0.001); (**c**) Highly significant differences in mesor and acrophase (*p* < 0.001) and a significant difference in amplitude (*p* < 0.05) between the LD12:12 group and LD16:8 group; (**d**) Highly significant differences in mesor and amplitude between the DD group and LD8:16 group (*p* < 0.001), with no significant difference in acrophase (*p* > 0.05); (**e**) Highly significant differences in mesor and amplitude between the LD12:12 group and LD8:16 group (*p* < 0.001), with no significant difference in acrophase (*p* > 0.05); (**f**) Highly significant differences in mesor, amplitude, and acrophase between the LD16:8 group and LD8:16 group (*p* < 0.001).

Inter-group rhythmic differences are illustrated in Figure 4 and Table 2. No significant differences were observed in mean brightness change rhythms between the DD and LD12:12 groups. However, the LD16:8 group exhibited highly significant differences in mesor and acrophase, along with significant amplitude differences compared to LD12:12. Specifically, extended photophase in LD16:8 reduced the mesor by 1.415, decreased amplitude by 0.988, and induced a 1.510 h acrophase delay. In contrast, the LD8:16 group showed highly significant differences in the mesor and amplitude relative to LD12:12, with no acrophase difference. The extended scotophase in LD8:16 increased the mesor by 2.141 and amplitude by 2.919. Compared to the DD group, LD16:8 demonstrated a highly significant rhythmic difference, with a 1.777 reduction in the mesor, a 1.847 decrease in amplitude, and a 1.508 h acrophase delay. The LD8:16 group exhibited highly significant differences in the mesor and amplitude relative to DD, but no acrophase difference. Specifically, the LD8:16 group had an increase in the mesor by 1.778 and amplitude by 2.061 compared to the DD group. Between LD16:8 and LD8:16, highly significant differences were observed, with LD16:8 displaying a 3.556 reduction in the mesor, a 3.908 decrease in amplitude, and a 1.638 h acrophase delay compared to LD8:16. These comparative results demonstrate that distinct photoperiods modulate rhythm parameters (mesor, amplitude, and acrophase) of body color change in juvenile *A. davidianus*, synchronizing their body color change cycles with external light–dark cues.

## 4. Discussion

### 4.1. Characteristics and Mechanisms of Circadian Rhythm of Body Color Change in Juvenile A. davidianus

The circadian rhythm, serving as a vital endogenous mechanism for organisms to synchronize with the Earth’s rotation, not only regulates physiological metabolism and behavioral patterns but also evolutionarily molds organisms to align with environmental light–dark cycles. As the primary zeitgeber, the photoperiod dynamically modulates circadian parameters (mesor, amplitude, and acrophase) by entraining endogenous circadian clocks, thereby optimizing survival strategies [18]. By elucidating the photoperiodic regulation of circadian rhythm of body color change in juvenile *A. davidianus*, this study demonstrates the synergistic interaction between endogenous circadian systems and exogenous photoperiodic signals in driving dynamic body color adaptation.

Juvenile *A. davidianus* exhibited distinct diurnal divergence in dorsal reflectance spectra and colorimetric variables. During the photophase, colorimetric variables including mean brightness (B2), green (S1G), yellow (S1Y), and red (S1R) were significantly lower than during the scotophase, while ultraviolet (S1U), violet (S1V), and blue (S1B) showed the opposite pattern. This body color shift manifested as dark brown color in the photophase and light brown color in the scotophase. The photophase spectra displayed similar flattened profiles with substantial overlap, and the colorimetric variables showed no significant temporal variations, indicating stable color during daytime. This stability likely reflects a strong photo-responsive mechanism maintaining a darker color under illumination. In contrast, the scotophase spectra exhibited pronounced undulations and greater dispersion, with significant temporal variations in colorimetric variables. Such nocturnal body color changes suggest that juvenile *A. davidianus* may utilize a gradual color shift to gauge nocturnal duration in the absence of photic cues.

Juvenile *A. davidianus* exhibited robust circadian rhythms in body color change, becoming darker during the photophase and lighter during the scotophase. Photoperiodic alterations significantly modulated the rhythm parameters, mesor (base level), amplitude (fluctuation intensity), and acrophase (peak time), thereby synchronizing body color change cycles with external light–dark cues. This synchronization reflects coordinated regulation by endogenous circadian clocks and exogenous photoperiodic signals, which is the core strategy for organisms to adapt to environmental changes, reflecting the flexibility and evolutionary advantages of the biological clock. The DD and LD12:12 groups displayed similar rhythmic patterns, indicating that the circadian rhythm of body color change in juvenile *A. davidianus* is endogenous and self-sustaining, regulated by an intrinsic circadian clock [45]. In contrast, the LD16:8 group exhibited a distinct rhythmic pattern compared to LD12:12, the extended photophase of which induced a 1.510 h acrophase delay and reduced the mesor and amplitude. This suggests a strong responsiveness to prolonged light exposure, with body color shifts initiating after light termination at 23:00, and mean brightness declining before reaching maximal levels. The LD8:16 group also diverged from LD12:12, showing an elevated mesor and amplitude without significant acrophase shifts. This indicates that juvenile *A. davidianus* exhibit a delayed response to an extended scotophase, with no significant lightening of body color observed within 2 h after light termination at 15:00, but body color shifts likely initiated between 17:00 and 19:00. The DD and LD12:12 groups exhibited analogous rhythmic patterns in body color, resulting in similar rhythm differences when compared to the LD16:8 and LD8:16 groups. Strikingly, the LD16:8 and LD8:16 groups displayed markedly distinct rhythmic patterns: an extended photophase failed to further decline mean brightness during light phases, whereas an extended scotophase significantly elevated mean brightness during dark phases. Compared to the American bullfrog (*Rana catesbeiana*) [15] and yellow cururu toad (*Rhinella icterica*) [26], juvenile *A. davidianus* demonstrated a more pronounced, stable, and flexible circadian rhythm of body color change. The rhythm integrates endogenous anticipation and exogenous plasticity—enabling predictive adjustments via internal circadian clocks to minimize zeitgeber-dependent lag costs while simultaneously tracking environmental cues to dynamically optimize the physiological and behavioral states for enhanced adaptability [46,47,48].

The circadian rhythm of body color change in juvenile *A. davidianus* is characterized by rapid and periodic change, belonging to a physiological color change that likely involves both primary and secondary color responses [49]. This process is regulated by biological oscillator systems [19,25] and mediated through photoperiod-dependent secretion of α-Melanocyte-Stimulating Hormone (α-MSH) and rhythmic melatonin release, which modulates melanocyte activity by inducing aggregation or dispersion of melanosomes [5,50,51]. These hormonal dynamics alter the skin’s capacity to absorb visible light, particularly long-wavelength red, yellow, and green light, resulting in either an overall increase in reflectance (manifested as brightness elevation and lightening) or a decrease (manifested as brightness reduction and darkening) [52]. This intricate mechanism underscores the interplay of multiple physiological pathways and environmental factors.

Observation revealed more pronounced and stable body color changes in juvenile *A. davidianus* during summer compared to winter, with a darker daytime body color in summer than winter. This seasonal plasticity may be attributable to lower winter temperatures inducing a hibernation-like state, which likely suppresses the circadian rhythm of body color change and a higher level of dark body color. Under controlled laboratory conditions with stable illumination (constant light intensity and full-spectrum composition), captive-bred juvenile *A. davidianus* exhibited a pronounced and stable circadian rhythm in body color change. However, this experimental design overlooked the dynamic photic variations inherent to natural habitats. Specifically, natural diurnal cycles are characterized by significant light intensity and spectral composition fluctuations. During dawn/dusk transitions, light intensity gradually increases/decreases, accompanied by a progressive spectral shift toward longer wavelengths (predominantly red/orange). At midday, light intensity peaks with a spectral bias toward shorter wavelengths (blue), while night-time exhibits minimal intensity and reduced ultraviolet (UV) or short-wavelength components, approximating darkness. Additionally, environmental stochasticity, such as canopy-induced light flecks and weather-driven light intensity variations, introduces irregular fluctuations in natural light regimes [53]. Consequently, compared to these dynamic natural conditions, simplified laboratory lighting may overestimate the oscillation amplitude and stability of circadian body color change, neglecting the critical regulatory role of spectral dynamics and limiting direct ecological extrapolation [54]. Notably, our experimental paradigm cannot resolve whether the observed rhythm represents evolutionary adaptations or phenotypic plasticity in response to artificial photic simplification. To enhance ecological relevance, future studies should adopt dynamic lighting systems to simulate latitude-specific solar trajectories and twilight-associated spectral transitions, integrate modulated light intensity programs replicating natural irradiance gradients and stochastic fluctuations, and validate findings through experiments in independent open-air field enclosures where juvenile *A. davidianus* are exposed to ambient environmental variables (e.g., diurnal light cycles, temperature fluctuations) while maintaining physical containment. This dual-validation framework will delineate the oscillatory characteristics of circadian body color change in juvenile *A. davidianus* under natural-light regimes, and further elucidate how circadian regulation of body color change balances intrinsic rhythmic stability and adaptive plasticity to photic complexity.

### 4.2. Function of Circadian Rhythm of Body Color Changes in Juvenile A. davidianus

The circadian rhythm of body color change in animals is hypothesized to serve multiple adaptive functions, including UV protection, energy conservation, camouflage, and thermoregulation. Among these, the UV protection hypothesis is considered the most prominent [27,55,56]. Both ultraviolet (UV) and visible light can trigger melanophore responses, but UV radiation carries significantly higher energy, capable of inducing lethal and sublethal effects at the organismal level [57]. During daylight hours of intense UV exposure, melanin dispersion absorbs UV radiation effectively, reducing its transmission to deeper tissues. This mechanism has been extensively validated in amphibians, where pigment granules (melanosomes) disperse under UV exposure, leading to skin darkening [58,59,60]. Supporting this, Liang (2015) documented that newly emerged larvae *A. davidianus* transitioned gradually from flesh-toned to dark black upon initial light exposure, with body color shifts accelerating under stronger illumination, suggesting light-induced melanin dispersion or synthesis [40]. Furthermore, the *A. davidianus*’s photophobic behavior, such as hiding beneath rocks or in caves, aligns with this adaptive strategy. Thus, daytime darkening in juvenile *A. davidianus* likely functions to mitigate UV-induced damage.

Nocturnal lightening of body color, characterized by melanin aggregation, may reflect an energy-saving strategy. As UV radiation diminishes at night, maintaining dispersed melanin becomes non-essential, and sustaining body color change incurs physiological costs. In melanophores, molecular motors utilize ATP hydrolysis to transport pigment granules [3,61]. While no direct evidence confirms greater energy expenditure during dispersion versus aggregation, daytime darkening likely requires more energy due to two processes: short-range melanosome dispersion and long-range pigment transfer from melanophores to keratinocytes [61]. Empirical evidence supports the metabolic cost of color change. Larvae of the Bosca’s newt (*Lissotriton boscai*) developed darker pigmentation on black backgrounds over two weeks, exhibiting higher metabolic rates compared to those depigmented on white backgrounds, confirming physiological costs associated with melanin production [62]. Indirect evidence further suggests that body color change is energetically demanding [27]. Species with rapid body color change rarely maintain maximal camouflage unless predator threats escalate [63], while slower-changing taxa preferentially occupy microhabitats matching their baseline color to optimize concealment [62,64,65]. Thus, nocturnal lightening in juvenile *A. davidianus* may optimize energy allocation while balancing photoprotection needs.

In contrast to the circadian rhythm of body color change observed in juvenile *A. davidianus*, the horn-eyed ghost crab (*Ocypode ceratophthalmus*) exhibits an inverse pattern: lightening to yellowish hues during daytime and darkening to gray-black tones at night [24]. This reverse rhythm may enhance daytime camouflage by matching sand color and brightness while optimizing nocturnal concealment in darker environments. Similarly, the variable seaweed shrimp (*Hippolyte varians*) displays a circadian rhythm of body color change—adopting green or brown tones matching daytime surroundings and transitioning to translucent pale blue at night [56,66]. These adaptations likely reduce detection by visual predators during daytime and improve concealment in deep-sea or dimly lit environments at night. In this study, juvenile *A. davidianus* darkened during the photophase, resembling the dark substrates of their natural habitats—a strategy potentially minimizing detection by daytime predators (e.g., birds). However, the adaptive value of nocturnal lightening remains ambiguous. Its efficacy may depend on complex factors, including low-light nocturnal conditions, aquatic activity depth, and predator sensory modalities, warranting further investigation.

Body color change plays a significant role in thermoregulation. Melanin aggregation (or reduction) under elevated temperatures reduces solar radiation absorption, whereas melanin dispersion (or increase) under cooler conditions enhances heat uptake [14,67]. However, this thermoregulatory function may not sufficiently explain the circadian rhythm of body color change, as daytime lightening—while thermally advantageous—could compromise UV protection. Thus, temperature-driven body color change likely holds explanatory power only for species exhibiting body color shifts exclusively during daytime. In the present study, smaller temperature variation and negligible diurnal thermal fluctuations were observed, suggesting that juvenile *A. davidianus* are unlikely to modulate body color primarily for thermoregulatory purposes.

## 5. Conclusions

This study presents the first systematic investigation of circadian body color change in juvenile *A. davidianus*, revealing statistically significant diurnal–nocturnal color shift characterized by daytime darkening and nocturnal lightening. The rhythm is regulated through coordinated interaction between endogenous circadian systems and exogenous photoperiodic cues, with greater responsiveness to photic than scotophasic stimuli. The observed body color change likely serves dual adaptive functions: ultraviolet (UV) radiation protection during the photophase and energy conservation during the scotophase. These findings lay critical groundwork for elucidating the physiological mechanisms and ecological significance of the circadian rhythm of body color change in this evolutionarily pivotal species.

## Figures and Tables

**Table 1:** Circadian rhythms of mean brightness variation under different photoperiod groups.

Photoperiodic	Rhythmic *p*	Mesor	Amplitude	Acrophase (h)	Peak Time
LD12:12 (*n* = 10)	0.000 ***	20.395 ± 1.167	7.600 ± 0.989	15.905 ± 0.355	00:54
DD (*n* = 10)	0.000 ***	20.758 ± 1.023	8.459 ± 1.165	15.908 ± 0.462	00:54
LD16:8 (*n* = 10)	0.000 ***	18.980 ± 0.622	6.612 ± 0.594	17.416 ± 0.443	02:25
LD8:16 (*n* = 10)	0.000 ***	22.536 ± 0.873	10.520 ± 1.182	15.778 ± 0.211	00:47

Note: Rhythmic *p* indicates the significance of circadian rhythm, where *** *p* < 0.001 indicates highly significant rhythmicity.

**Table 2:** Differences in circadian rhythms of mean brightness variation between photoperiod groups.

Group Comparison	Mesor diff.	*P* (Mesor diff.)	Amplitude diff.	*P* (Amplitude diff.)	Acrophase diff.	*P* (Acrophase diff.)
DDvsLD12:12	0.363	0.283	0.859	0.081	0.002	0.980
LD16:8vsLD12:12	−1.415	0.000 ***	−0.988	0.020 *	1.510	0.000 ***
LD8:16vsLD12:12	2.141	0.000 ***	2.919	0.000 ***	−0.128	0.428
LD16:8vsDD	−1.777	0.000 ***	−1.847	0.000 ***	1.508	0.000 ***
LD8:16vsDD	1.778	0.000 ***	2.061	0.000 ***	−0.130	0.508
LD16:8vsLD8:16	−3.556	0.000 ***	−3.908	0.000 ***	1.638	0.000 ***

Note: * *p* < 0.05 indicates significant difference; *** *p* < 0.001 indicates highly significant difference.

## Data Availability

The data presented in this study are available within this article.
